# Research Progress and the Prospect of Artificial Reef Preparation and Its Impact on the Marine Ecological Environment

**DOI:** 10.3390/ma19030447

**Published:** 2026-01-23

**Authors:** Hao-Tian Li, Ya-Jun Wang, Jian-Bao Zhang, Peng Yu, Yi-Tong Wang, Jun-Guo Li, Shu-Hao Zhang, Zi-Han Tang, Jie Yang

**Affiliations:** College of Metallurgy and Energy, North China University of Science and Technology, 21 Bohai Street, Tangshan 063210, China; 18032815354@163.com (H.-T.L.); wangyj@ncst.edu.cn (Y.-J.W.); zhangjianbao@ncst.edu.cn (J.-B.Z.); 18987542339@163.com (P.Y.); psyzhang7@163.com (S.-H.Z.); lisatzh4309@163.com (Z.-H.T.); wangcong@ncst.edu.cn (J.Y.)

**Keywords:** artificial reef, new concrete material, ecological restoration, fishery economy

## Abstract

Artificial reefs are an important tool for marine ecological restoration and fishery resource proliferation, and are widely used around the world. Among them, Japan, the United States, China, South Korea, Australia, and the Mediterranean coastal countries have particularly invested in scientific research and practice in this field, and the reefs’ material selection, structural performance, and ecological benefits have attracted much attention. The purpose of this paper is to summarize the preparation methods, characterization methods (such as microstructure analysis and mechanical tests) and mechanical properties (such as compressive strength and durability) of new concrete materials (steel slag-blast furnace slag concrete, oyster shell concrete, sulfoaluminate cement concrete, recycled brick concrete, silica fume concrete, and banana peel filler concrete) that artificial reefs and ceramic artificial reefs developed in recent years, and to explore the resource utilization potential of different waste materials. At the same time, the biostatistical methods (such as species abundance and community diversity) of wood, shipwreck, steel, rock, waste tire, and ordinary concrete artificial reefs and their effects on the marine environment were compared and analyzed. In addition, the potential impact of artificial reef deployment on local fishermen’s income was also assessed. It is found that the use of steel slag, blast furnace slag, sulfoaluminate cement, and silica fume instead of traditional Portland cement can better improve the mechanical properties of concrete artificial reefs (compressive strength can be increased by up to 20%) and reduce the surface pH to neutral, which is more conducive to the adhesion and growth of marine organisms. The compressive strength of oyster shell concrete and banana peel filler concrete artificial reef is not as good as that of traditional Portland cement concrete artificial reef, but it still avoids the waste of a large amount of solid waste resources, provides necessary nutritional support for aquatic organisms, and also improves its chemical erosion resistance. The deployment of artificial reefs of timber, wrecks, steel, rock, waste tires, and ordinary concrete has significantly increased the species richness and biomass in the adjacent waters and effectively promoted the development of fisheries. Cases show that artificial reefs can significantly increase fishermen’s income (such as an increase of about EUR 13 in the value of a unit effort in a certain area), but the long-term benefits depend on effective supervision and community co-management mechanisms. This paper provides a scientific basis for the research and development of artificial reef materials and the optimization of ecological benefits, and promotes the sustainable development of marine ecological restoration technology and fishery economy.

## 1. Introduction

Marine ecosystems are facing increasingly severe challenges, and the fishing of marine organisms has led to a sharp decline in fishery resources [1] and increased emissions of petroleum hydrocarbons and heavy metals; additionally, other pollutants near the coastline have seriously damaged coastal ecosystems and reduced biodiversity [2,3]. As an ecological restoration method and artificial underwater structure, artificial reefs are widely used in the restoration of coastal ecosystems and fishery resources [4]. Artificial reefs have been applied in at least 40 countries [5]. In particular, Japan, the United States, China, South Korea, Australia, and the Mediterranean countries in the field of scientific research and practice investment are particularly prominent. By simulating natural reef structures, artificial reefs provide habitats, breeding, and foraging sites for marine organisms, increase commercial fishery production, and improve the damaged seabed, thereby achieving the purpose of increasing fishery resources and improving the marine environment [6,7,8,9].

Concrete is the most widely used material for the construction of artificial reefs. It can be used to manufacture structures with complex shapes and large hollow shapes [10]. However, traditional concrete artificial reefs have high pH and poor biological adhesion, and there are problems such as large resource consumption and environmental pollution in the process of production and use [11]; the development of environmentally friendly artificial reef materials has become a research hotspot. In recent years, a variety of new concrete materials, such as steel slag-blast furnace slag concrete, oyster shell concrete, sulfoaluminate cement concrete, recycled brick concrete, and silica fume concrete, because of their excellent mechanical properties and environmental characteristics [12], show great application potential in the field of artificial reefs. Olatoyan et al. [13] prepared green concrete reefs with steel slag instead of natural coarse aggregate, and their compressive strength, impact resistance, and chemical erosion resistance are better than those of traditional granite aggregate. Meng et al. [14] used oyster shells as aggregates to prepare concrete reefs. The porosity of oyster shells can absorb water and release it during curing, providing internal curing for concrete and helping to improve the early strength of reefs. The concrete reef prepared by Li et al. [15] using sulfoaluminate cement as gel material has good thermal insulation, compressive strength, and waterproof performance. These materials can not only effectively utilize industrial waste and marine resources and reduce environmental load, but also provide a more suitable living environment for marine organisms.

Traditional materials such as wood, shipwrecks, ceramics, waste tires, steel, and rock also have unique structures and ecological functions [10]. They play an important role in the construction of artificial reefs. Collins et al. [16] deployed an artificial reef made of waste tires, and found that tires release zinc and accumulate in some epiphytic organisms (such as hydra), but there is little difference in biological colonization between tires and concrete as a whole. Oren et al. [17] employed parametric design and 3D printing technology to fabricate complex ceramic fish reef structures. The intricate structures created through 3D printing successfully provided diverse habitats for organisms occupying different ecological niches. The Recfishwest team studied the stability of a variety of steel structures (water buoys, three-legged platforms, and single-pile platforms) in the sea and found that the water buoy is considered to be basically suitable, mainly because of its complex structure, good habitat, and relatively low risk of pollutants. The team pointed out that carbon steel has poor corrosion resistance in the marine environment, while stainless steel has better corrosion resistance due to the presence of molybdenum, but the specific performance depends on the steel grade [18]. Artificial reefs made from different materials exhibit significant variations in preparation techniques, structural properties, ecological effects, and environmental friendliness, necessitating systematic research and evaluation. Under the framework of circular economy, the above materials as artificial reefs not only realize the transformation of ‘waste to resources’, but also produce systematic value-added effects in environmental carbon reduction, ecological restoration, economic conservation, and other aspects. Its successful implementation also needs to be combined with scientific evaluation, engineering specifications, and ecological monitoring [19].

The purpose of this paper is to summarize the preparation methods, characterization methods, and mechanical properties of steel slag concrete, oyster shell concrete, sulfoaluminate cement concrete, recycled brick concrete, silica fume concrete, banana peel filler concrete, and ceramic artificial reefs. The biological statistical methods of wood, shipwreck, steel, rock, waste tires, and ordinary concrete artificial reefs and their impact on the marine environment are discussed, and the impact of the deployment of artificial reefs on the income of local fishermen is discussed. It provides a scientific basis for the development and application of artificial reef materials, and promotes the sustainable development of marine ecological restoration technology and the fishing industry.

## 2. Concrete Artificial Reef

### 2.1. Steel Slag-Blast Furnace Slag Concrete Artificial Reef

**Figure 1:**
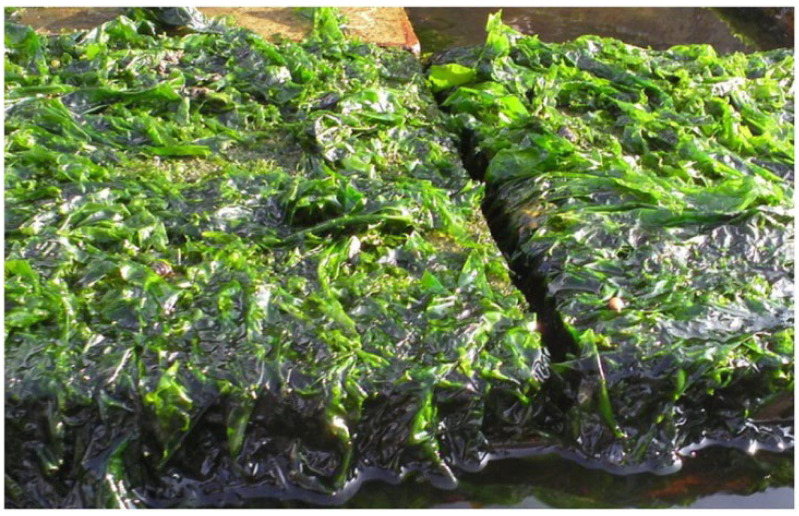
The attachment and growth of algae on concrete artificial reef specimens after 8 months in the sea [22].

Blast furnace slag is a by-product produced in the process of blast furnace ironmaking, which is mainly produced by the reaction of iron ore, coke, and limestone at high temperature. Steel slag is a solid waste generated during iron and steel smelting. It is mainly formed by impurities in raw materials, furnace materials, eroded furnace lining, and oxidized impurities. At present, the comprehensive utilization rate of blast furnace slag in China is about 80% [20], and its resource utilization is mainly concentrated in the large-scale utilization fields such as building materials, while the utilization rate of steel slag is less than 30%, which is far lower than that of developed countries [21]. A large number of steel slag and blast furnace slag have caused serious environmental problems and economic losses. However, steel slag and blast furnace slag can be used as raw materials for the preparation of green artificial reef concrete [22]. This will conserve natural resources and lower material expenses, which benefits the sustainable growth of both the iron and steel industry and the fishery sector. Li et al. [23] used a four-factor three-level orthogonal design to investigate the effects of the ratio of steel slag to blast furnace slag, steel slag grinding time, water-binder ratio, and superplasticizer dosage on the compressive strength of concrete. The specimens were subjected to standard curing and simulated seawater curing, and the hydration products and microstructure were analyzed by X-ray diffraction (XRD) and scanning electron microscopy (SEM). It was found that when the ratio of steel slag to blast furnace slag was 5:3, the grinding time of steel slag was 90 min, the water-binder ratio was 0.20, and the amount of water reducing agent was 0.2%; the strength of the cementitious material was optimal. The compressive strength of the concrete block prepared under this condition exceeds 50 MPa after 28 days of standard curing, which meets the requirements of high-strength artificial reefs. XRD and SEM analysis showed that the early hydration products were mainly the ettringite phase and C-S-H gel, while the later strength development was primarily attributed to the pozzolanic reaction of the steel and blast furnace slag. The strength of the test block under simulated seawater curing conditions is slightly higher than that under standard curing conditions, indicating that steel slag concrete has a good application prospect in the marine environment. Huang et al. [22] prepared gel materials with blast furnace slag, steel slag, cement clinker, and desulfurized gypsum according to the ratio of 381.4:54.5:54.5:54.5. Steel slag was used as fine and coarse orthopedics, and artificial reefs were prepared with cementitious materials, steel slag fine aggregate, steel slag coarse aggregate, and a water reducer ratio of 544.9:1330.5:785.7:1.64. The hydration products of steel slag-blast furnace slag concrete artificial reefs were studied by XRD, differential scanning calorimetry (DSC), and SEM analysis. In order to compare, a typical Portland cement slurry was prepared using the same amount of water and superplasticizer as the steel slag-blast furnace slag concrete slurry. The results showed that the compressive strength of steel slag-blast furnace slag concrete reached 71.4 MPa at 28 days, and the compressive strength was 92.5 MPa after 240 days of additional curing in artificial seawater. Through XRD analysis and the comparison of steel slag-blast furnace slag concrete slurry on day 3 and day 28 with typical Portland cement slurry on day 28, it is found that the hydration products of steel slag-blast furnace slag concrete are C-S-H gel and ettringite, and a considerable amount of silicate is not detected. This is due to the minimal amount of cement clinker in the slurry, and the absence of silicate in the paste’s hydration products. This is desirable for artificial reefs because it is beneficial to reduce their pH to prevent pH-resistant organisms, such as barnacles, from attaching to the surface of concrete artificial reefs, as shown in Figure 1, thereby enabling the settlement of more productive marine organisms such as algae.

**Figure 2:**
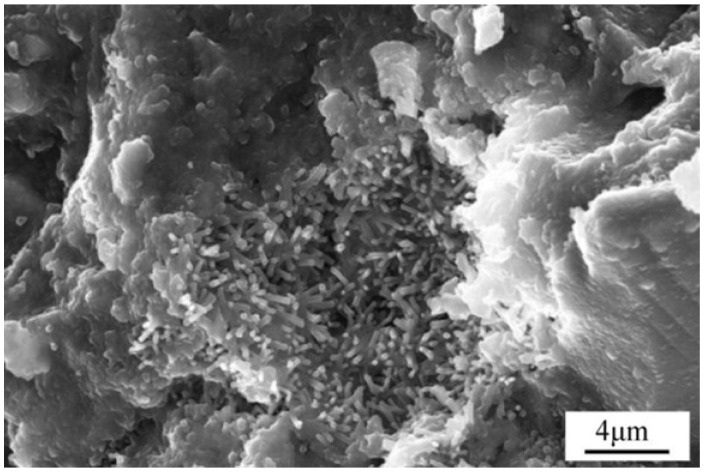
SEM images of steel slag concrete slurry on the 28th day [22].

The results of DSC analysis were consistent with those of XRD analysis, and no considerable amount of silicate was detected. Through SEM analysis, it is observed that the very dense C-S-H gel structure in the steel slag-blast furnace slag concrete sample is observed in Figure 2, which ensures its high compressive strength.

Zhao et al. [24] used magnetic steel slag powder and steel slag mud to mix with water for compression molding, followed by carbonization and curing in simulated seawater. Through compressive testing, XRD, thermogravimetric analysis, and SEM analysis, it was found that carbonation and hydration reaction occurred simultaneously in the solidification of steel slag powder, while carbonation reaction was the main reaction in steel slag mud. During seawater curing, the strength of steel slag powder samples increases first due to the formation of hydration products, and then gradually decreases and stabilizes with its decomposition. The strength of the steel slag mud sample decreased first and then increased, and recovered due to the conversion of calcite to vaterite, and finally stabilized. Studies have shown that the durability of materials in seawater depends on the CaO content in carbonated active minerals. Steel slag mud is more suitable for the preparation of artificial reefs because of its stronger corrosion resistance. The research findings indicate that artificial reef concrete made from steel slag and blast furnace slag can achieve compressive strengths exceeding 50 MPa, with strength continuing to increase in seawater. Its surface, characterized by low alkalinity and specific hydration products (primarily C-S-H and calcium aluminate hydrate), promotes algal attachment. The dense microstructure ensures low porosity and high durability. This technology effectively achieves high-value resource utilization of metallurgical solid waste, delivering both environmental and economic benefits. Furthermore, carbonation and the pozzolanic reaction of blast furnace slag enhance its mechanical properties, meeting the requirements for high-strength artificial reefs.

### 2.2. Oyster Shell Concrete Artificial Reef

**Figure 3:**
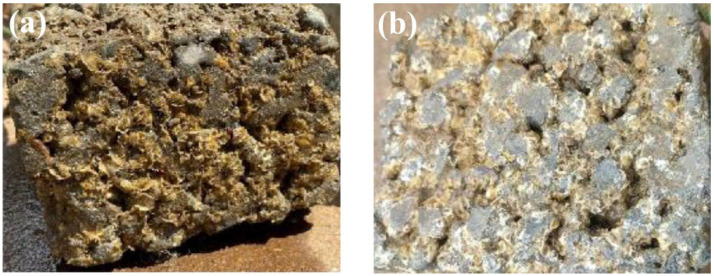
Biological attachments of artificial reefs after 1 month: (**a**) Oyster shell content is 20%. (**b**) Oyster shell content is 0% [29].

The world’s oyster farming and food service sectors have generated over 3 million tons of waste oyster shells in 2024 [25], occupying valuable tidal flat resources. At the same time, the organic and inorganic substances in the discarded oyster shells will gradually decompose and release a large amount of ammonia nitrogen, phosphate, and other harmful substances in the process of abandonment, resulting in environmental pollution problems such as eutrophication and deterioration of water quality [26]. Relevant studies have found that the main component of an oyster shell is CaCO_3_. If discarded, it will cause a lot of solid waste resources to be wasted. It can be used as an inert filler to prepare concrete artificial reefs. Mohammad et al. [27] used waste oyster shells as inert fillers to prepare permeable oyster shell habitat concrete, which has the advantages of low cost, good durability, and low carbon footprint. Kuo et al. [28] used waste oyster shells to replace sand in concrete, and the reduction in compressive strength was only less than 20%. Concrete serves as the primary material for the construction of artificial reefs [10]. Kong et al. [29] prepared porous concrete artificial reefs with a ratio of cement, natural aggregate, and water of 559:1598:168, and used waste oyster shells as insertion fillers to replace 0%, 20%, and 40% of cement (by mass). The interconnected porosity of the oyster shell concrete artificial reef was tested, and the compressive strength, sulfate dry–wet cycle, sulfate corrosion resistance, and leaching solution alkalinity were tested for different curing days. The results show that the average difference between the actual porosity and the designed interconnect porosity is only 0.5, indicating that the recycled aggregate, waste oyster shell, and cement slurry in concrete have excellent compatibility. The smooth surface of oyster shell powder hinders the close adhesion of C-S-H gel. Therefore, as the replacement rate of recycled aggregate and waste oyster shell increases, a weak interface develops around the oyster shell powder, diminishing the compressive strength. However, its compressive strength still meets the Chinese fishery industry standard SC-T9416-2014 [30], and it can resist the sulfate erosion test no more than 90 times, which meets the requirements of KS90 in national standard GB-T 50082-2009 [31]. Due to the porous structure of porous concrete, ettringite and gypsum, produced in the early stage of sulfate attack, fill the pores, so that the concrete has higher strength, but the size of gypsum and ettringite will expand with the increase in the dry–wet cycle, and the destruction of the structure will eventually lead to the generation of cracks and the loss of adhesion [32]. The alkalinity primarily results from the liberation of hydroxyl ions during cement hydration [33]. Leachate pH decreases with the increase in recycled aggregate and waste oyster shell replacement rate, which is more conducive to marine biological attachment [34], as shown in Figure 3.

Rupasinghe et al. [35] prepared oyster shell concrete artificial reefs with cement and blast furnace slag as gel materials, river sand, and sea sand as fine aggregate, and oyster shell as coarse aggregate. The compressive strength test, material surface pH test, and carbonization test were carried out on the configured oyster shell concrete artificial reef. The results show that the compressive strength of the oyster shell concrete artificial reef rises progressively as curing time extends. Because the chloride concentration in sea sand exceeds that in river sand, the additional chloride ions accelerate the reaction between the aluminum-rich phases in the blast furnace slag, resulting in a higher compressive strength of the mixture of sea sand than that of river sand [36]. Alkalinity primarily results from the liberation of hydroxyl ions during cement hydration. Cement hydration releases hydroxyl ions, while blast furnace slag consumes hydroxyl ions through the pozzolanic reaction. Compared with the completely cement-based mixture, blast furnace slag-based concrete significantly reduces the alkalinity of the material, which is more conducive to marine biological attachment. Through carbonation tests, carbonation serves as an effective technique for lowering the pH of oyster shell concrete artificial reefs, notably in blast furnace slag-based mixtures. Carral et al. [37] prepared concrete artificial reefs using oyster shells as fine aggregate and medium aggregate, respectively. The prepared oyster shell concrete artificial reef was subjected to a compression test, XRD analysis, and SEM analysis. The results show that the compressive strength of concrete artificial reefs with oyster shells as fine bones for 7 days and 28 days is 30.9 MPa and 35.8 MPa, respectively. The compressive strength of concrete artificial reefs with oyster shells as medium bones for 7 days and 28 days is 34.3 MPa and 39.4 MPa, respectively. With the increase in curing time and aggregate particle size (from fine particle size to medium particle size), the compressive strength of oyster shell concrete artificial reefs gradually increases. Through XRD analysis, it was found that the calcium carbonate in the oyster shell was presented in the form of calcite, and the incorporation of calcite helped to form a dense calcium carbonate protective layer on the surface of the concrete to prevent chemical erosion from seawater [38,39]. Through SEM analysis, it was observed that the oyster shell particles in the artificial reef showed a layered structure, which reduced the adhesion between the cement and oyster shell and increased the porosity of concrete. As a result, the overall mechanical properties of oyster shell concrete artificial reef were not as good as those of traditional concrete artificial fish. Cohen et al. [40] prepared oyster shell concrete artificial reef with a ratio of cement, oyster shell, and water of 525:170:85 and tested its ability to promote ammonium oxidation. The results showed that the concentration of NH_4_-N in the soaking solution of oyster shell concrete artificial reef decreased significantly over time, which reduced the negative impact of fish growth and reproduction. The artificial reef made of oyster shell and cement showed the ability to promote ammonium oxidation similar to natural reefs. Research indicates that artificial reefs made from concrete using discarded oyster shells exhibit slightly reduced compressive strength compared to traditional concrete (decrease < 20%), yet still meet industry standards. Their porous structure and suitable surface texture promote biological attachment, while incorporating slag or carbonation treatment effectively lowers surface alkalinity, making them more adaptable to marine environments. This material achieves resource utilization of oyster shell waste while offering low cost and environmental friendliness. It generally meets construction requirements for artificial reefs in terms of mechanical properties, pore structure, and ecological compatibility.

### 2.3. Recycling Brick Aggregate Concrete Artificial Reef

Accelerated urbanization has resulted in a marked growth in construction waste generation, with 4.5 billion tons of debris produced solely in China by 2024 [41]. The massive accumulation of construction waste has caused serious environmental pollution and waste of resources [42,43]. Relevant studies have found that the main chemical components of recycled bricks are SiO_2_, Al_2_O_3_, and Fe_2_O_3_, which can be used as raw materials to prepare artificial reefs to reduce their material costs and reduce environmental resource consumption [44,45]. Wang et al. [46] prepared concrete artificial reefs with recycled bricks as aggregates, Portland cement and sulfoaluminate cement as gel materials, and fly ash and silica fume as mineral additives. After a thorough mixing of all components, the slump test was conducted to assess the slurry’s workability; the mixture was then poured into a mold wrapped in plastic sheeting and cured for 24 h. The compressive strength, drying shrinkage, and chloride resistance of artificial reefs with varying curing durations were assessed. The results show that the slump of the slurry does not exceed 70 mm, which meets the standard requirements. With the passage of time, the compressive strength of concrete artificial reefs gradually increases. The reaction of silicate and calcium ions in water forms an appropriate amount of calcium silicate hydrate, which can promote the hydration of cement and improve the compressive strength [47]. The artificial reef prepared by sulfoaluminate cement, recycled brick and silica fume has the highest compressive strength, the lowest drying shrinkage, and the best resistance to chloride ion permeability. Silica fume can occupy tiny pores in concrete, thereby enhancing compactness and density. Compared with Portland cement, sulfoaluminate cement greatly enhances the resistance to drying shrinkage, and the addition of silica fume and fly ash is also conducive to enhancing the resistance to drying shrinkage so that artificial reefs have higher quality and service life. Sulfoaluminate cement exhibits a strong ability to bind chloride ions, thereby reducing free chloride concentrations penetrating the material and enhancing erosion resistance.

### 2.4. Sulfoaluminate Cement, Sea Sand, and Seawater Concrete Artificial Reefs

As a high-strength cementitious material, sulfoaluminate cement has received extensive attention in marine engineering due to its unique properties. It exhibits excellent resistance to chloride ion penetration, making it have broad application prospects in the marine environment [48]. As sustainable alternative materials, seawater and sea sand have been widely used in concrete mixtures, especially in these resource-rich coastal areas [49]. The use of sulfoaluminate cement in combination with seawater and sea sand has shown enhanced mechanical properties and durability, which are essential for the long-term stability of artificial reefs [50]. Functioning as a crucial habitat for marine life, artificial reefs enhance biodiversity and facilitate the recovery of fishery resources [10]. Building artificial reefs with sulfoaluminate cement-based seawater sea sand concrete addresses shortages in conventional construction materials while aiding in restoring marine ecology [51]. Chen et al. [51] used scintillation as coarse aggregate, river sand and sea sand as fine aggregate, Portland cement and sulfoaluminate cement as gel materials, and added seawater and fresh water to prepare concrete artificial reefs. The mixing ratio is shown in Table 1.

**Figure 4:**
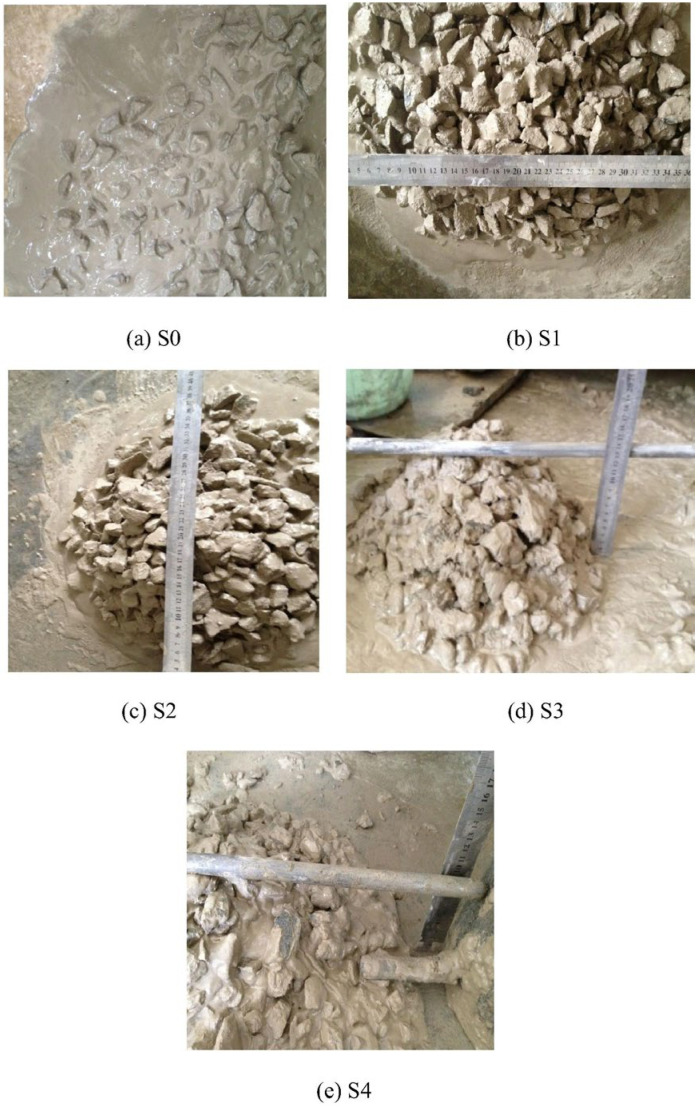
Cohesiveness and water retention of artificial reefs with different material ratios [51].

All materials were mixed evenly, and the slump, slump loss, and slump flow tests were first performed to determine the workability of the slurry, and then the mixture was poured into a mold with an oiled inner surface for compaction and curing for 24 h. The compressive strength, ultrasonic pulse velocity, concrete surface pH, pore structure analysis, XRD, and SEM tests were carried out on the solidified artificial reef. The results indicate a larger specific surface area for sulfoaluminate cement compared to Portland cement, and for sea sand relative to river sand. Under the same mixing ratio, the free water content in sulfoaluminate cement and sea sand slurry is low and the operability is strong [52,53], as shown in Figure 4, and the calcium chloride in seawater promotes the hydration of concrete [54].

**Figure 5:**
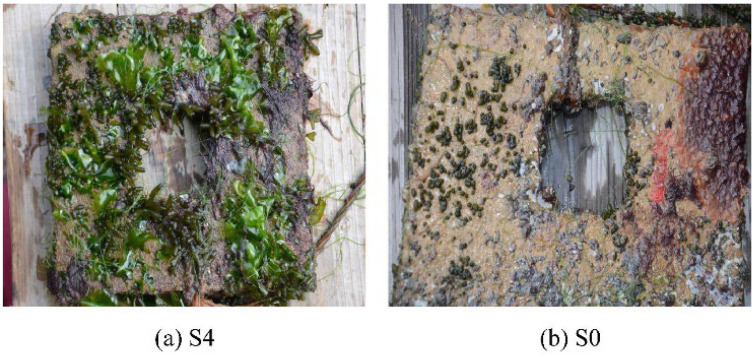
Growth of organisms on artificial reefs [51].

Through XRD and SEM analysis, it is found that the hydration products of sulfoaluminate cement make the concrete have a closer pore structure and interface transition zone, and the fineness modulus of sea sand is smaller. Sulfoaluminate cement can bind chloride ions from both sea sand and sea water to form Friedel’s salt, thereby filling inter-skeletal spaces [55]. The leaching rate of alkaline substances in concrete is lower, resulting in the best workability of sulfoaluminate cement-based concrete artificial reefs in their experiments, the maximum compressive strength of concrete, the lowest pH, and the highest amount of biological adhesion as shown in Figure 5.

Xu et al. [56] used scintillation as coarse aggregate, river sand and sea sand as fine aggregate, Portland cement and sulfoaluminate cement as gel materials, and added seawater and fresh water to prepare concrete artificial reefs. The mixing ratio is shown in Table 2.

All materials were mixed evenly. Initially, the slump and slump loss tests were conducted to evaluate the workability of the slurry; subsequently, the mixture was transferred into the mold and sealed with plastic film for 24 h. The compressive strength, concrete surface pH, pore structure analysis, XRD, and SEM tests were carried out on the solidified concrete artificial reef. The results show that the compressive strength of concrete artificial reefs prepared by sulfoaluminate cement is significantly higher than that of artificial reefs prepared by Portland cement, and the surface pH is lower, which is conducive to the rapid attachment of marine organisms. Due to its hydration, Friedel’s salt acts as a filler in concrete pores, enhancing the material’s pore structure, improving compactness, and preventing alkaline liquid leakage [57,58]. However, blending sulfoaluminate cement with Portland cement will markedly impair the workability and mechanical properties of artificial reefs, while the lessening effect on surface pH is not significant. Sea sand and seawater positively influence the mechanical characteristics of sulfoaluminate cement-based artificial reefs, particularly during early strength development. Sea sand and seawater improve the pore structure of concrete, reduce the pore volume and average pore size, and thus improve the compactness and durability of concrete. However, sea sand and seawater exert minimal influence on the surface pH of artificial reefs. Tao et al. [59] used scintillation as coarse aggregate, river sand and sea sand as fine aggregate, Portland cement and sulfoaluminate cement as gel materials, and added seawater and fresh water to prepare concrete artificial reefs. The mixing ratio is shown in Table 3.

**Figure 6:**
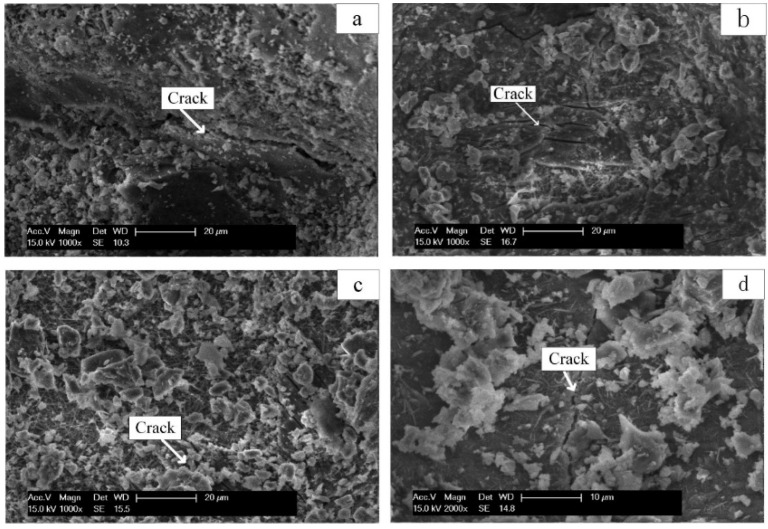
Microscopic state of internal cracks in artificial reefs. (**a**) OSM; (**b**) OOSM; (**c**) SSM; (**d**) OFR [59].

The microstructure, chloride content, chloride penetration, capillary water absorption, and anodic polarization of the solidified concrete artificial reef were tested. The results show that the sulfoaluminate cement-based artificial reef has a denser microstructure, fewer internal microcracks, better pore structure and chloride ion binding rate, and its chloride ion permeability and capillary water absorption are the lowest, indicating that it has better permeability and durability. The permeability and water absorption of the mixed cement-based artificial reef are the highest, indicating that its microstructure is poor as shown in Figure 6.

**Figure 7:**
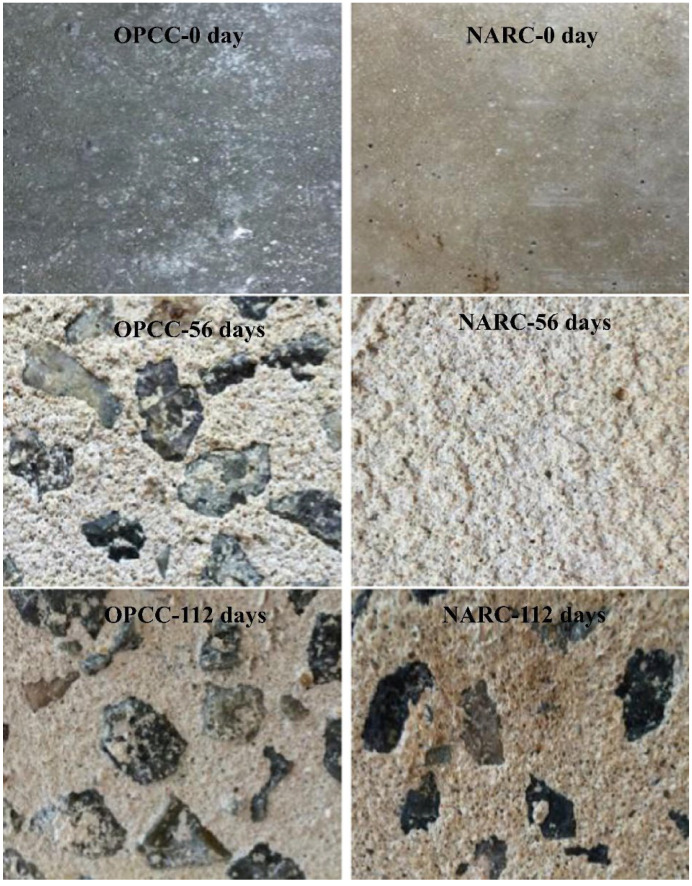
Surface images of NARC and OPCC attacked by biological sulfuric acid [58].

The corrosion degree of steel bars in sulfoaluminate cement-based artificial reefs is low, which is mainly attributed to the high chloride binding rate and dense microstructure of sulfoaluminate cement. However, due to the introduction of a large amount of chloride ions into sea sand and seawater, the corrosion of steel bars in sulfoaluminate cement-based artificial reefs is more significant than that of ordinary concrete reefs prepared with fresh water and river sand [60]. Adding 2% corrosion inhibitor can significantly reduce the corrosion degree of steel bars in sulfoaluminate cement-based artificial reefs. Yang et al. [58] studied the corrosion resistance of concrete artificial reefs prepared from sulfoaluminate cement, sea sand, and seawater under a biosulfuric acid corrosion environment, and compared it with concrete artificial reefs prepared from ordinary Portland cement, river sand, and fresh water. The mixing ratio is shown in Table 3. The surface morphology analysis, microstructure analysis, compressive strength test, and mass loss test of two kinds of concrete artificial reefs were carried out after simulating the biological sulfuric acid corrosion of the marine environment. The results show that, as shown in Figure 7, the surface roughness of sulfoaluminate cement-based artificial reefs increases slowly and the degree of corrosion is light under the corrosion of biological sulfuric acid, while the surface corrosion of Portland cement-based artificial reefs is more serious, which is manifested as surface whitening, mortar softening, and aggregate exposure.

**Figure 8:**
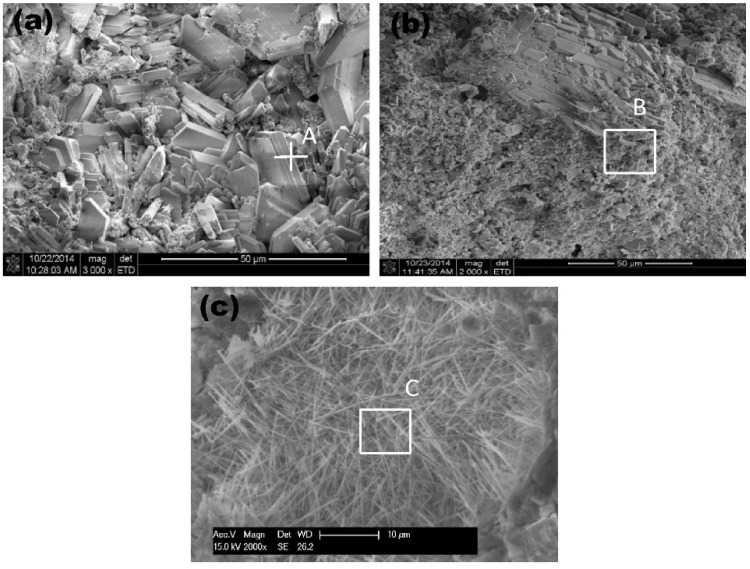
The morphology of NARC eroded by biosulfuric acid for 56 days in (**a**) near-surface zone, (**b**) transition zone, and (**c**) uncorroded zone [58]. Letters represent the positions where the EDS analysis of the material is carried out.

Through SEM analysis, it was found that under the corrosion of biological sulfuric acid, the hydration product ettringite of sulfoaluminate cement-based artificial reef was converted into gypsum, as shown in Figure 8.

**Figure 9:**
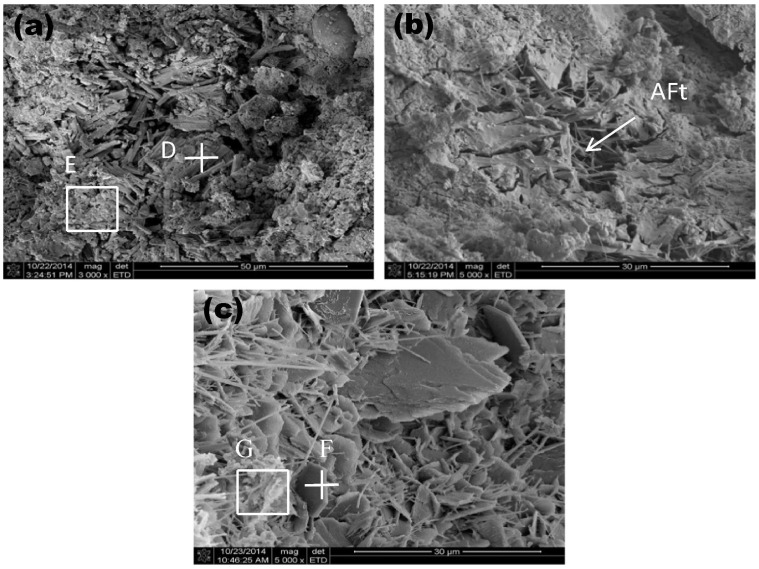
The morphology of OPCC eroded by biosulfuric acid for 56 days in (**a**) near-surface zone, (**b**) transition zone, and (**c**) uncorroded zone [58]. Letters represent the positions where the EDS analysis of the material is carried out.

The hydration products Ca(OH)_2_ and C-S-H of the cement-based artificial reef are converted into gypsum and non-bonded silica gel, resulting in an increase in porosity and accelerating the corrosion process [61] as shown in Figure 9.

The compressive strength loss rate and mass loss rate of sulfoaluminate cement-based artificial reefs are significantly lower than those of Portland cement-based artificial reefs. In summary, the aluminate cement-based artificial reef utilizes sustainable materials such as seawater and marine sand, exhibiting high compressive strength, low surface pH (favorable for biofouling), and low porosity (dense structure), while demonstrating slow alkali leaching. These characteristics significantly enhance mechanical stability and durability while supporting resource recycling. Overall, this material meets the requirements for long-term structural performance and ecological restoration in artificial reefs.

### 2.5. Silica Fume Concrete Artificial Reef

Traditional concrete is susceptible to corrosion and erosion in the marine environment, affecting its long-term stability. To enhance concrete performance, silica fume can be incorporated as an admixture in the construction of artificial reefs. Silica fume concrete exhibits high compressive strength, low permeability, and good durability, effectively resisting physical and chemical erosion in marine environments [62,63]. Nagalakshmi et al. [64] designed the mix ratio of concrete according to the IS standard [65] and poured the specimens. The specimens were dried for 1 day and cured for 28 days after pouring, and then the slump, compressive strength, tensile strength, and permeability were tested. In order to optimize the structural design of artificial reefs, the researchers used AutoCAD (v2016) and CATIA software (v5) to design a hemispherical reef structure, and carried out mechanical analysis on it through ANSYS software (v19.0). During the analysis, the hydrostatic pressure and hydrodynamic pressure of the reef at 15 m and 30 m water depth were simulated to evaluate its stability in different marine environments. The experimental results show that silica fume concrete exhibits excellent performance in the construction of artificial reefs. With the increase in silica fume content, the compressive strength, and tensile strength of concrete increase significantly, especially at 10% silica fume content where the mechanical properties of concrete reach their best. The impermeability of silica fume concrete is greatly diminished, suggesting its enhanced corrosion resistance. Through the analysis of ANSYS software, the researchers found that the designed hemispherical reef structure can withstand large water pressure at 15 m and 30 m water depth, showing good stability. The design of holes in the reef structure not only contributes to the passage of water, but also provides a space for marine organisms to inhabit and move. In summary, silica fume concrete, as a high-performance material, can effectively improve the durability and functionality of artificial reefs, and provide reliable technical support for the protection and restoration of marine ecosystems.

### 2.6. Banana Skin Filler Concrete Artificial Reef

A large amount of agricultural waste such as banana peel is produced every year in the world [66]. A large amount of accumulation will produce harmful substances such as bacteria and molds, which may pose a threat to the natural environment and human health. However, banana peel is rich in organic matter and crude fiber, which can be dried as a filler for the preparation of concrete artificial reefs (Jusoh et al. [67]). Different proportions of banana peel particles-recycled aggregate concrete cubes (10%, 20% and 30% banana peel particles) were prepared, and the pure recycled aggregate concrete cubes were used as the control group. After curing at room temperature for 28 days, the compressive strength of concrete cubes was tested to evaluate their mechanical properties. By simulating the wave environment, the contents of total nitrogen, total phosphorus, and total organic carbon released from artificial reefs after 6 days of immersion in water were tested to analyze the release behavior of nutrients. The results showed that the compressive strength of concrete decreased significantly with the increase in banana peel particle content. The cube compressive strength of 10% banana peel particles was 26.8 MPa, 20% was 18.3 MPa, and 30% was only 11.0 MPa (control group was 43.0 MPa). However, the addition of banana peel particles significantly increased the release capacity of nutrients, and the release of total nitrogen, total phosphorus, and total organic carbon reached 34.3~41.5%, 58.2~70.3%, and 7.2~10.6%, respectively.

## 3. Other Material Artificial Reefs

### 3.1. Wood Artificial Reefs

As a natural material, wood has a wide range of sources, good biocompatibility and degradability, and has good fish appeal [68]. It can provide a suitable habitat environment for marine organisms. Alam et al. [69] conducted a three-year field experiment in Sanjin Bay, Hiroshima, Japan. Three types of artificial reefs were designed in the experiment: simple timber reefs, timber reefs with oyster shells, and timber reefs with leaves/branches. Fifteen artificial reef structures were installed at two locations within Sanjin Bay, positioned at a depth of 10 m. The research team regularly collected marine biological samples attached to artificial reefs and identified species or lowest taxonomic units. The species diversity index, richness index, and evenness index were calculated to evaluate ecological diversity. The findings indicated that after the placement of artificial reefs, the number of marine organisms surged in the first year, and then declined steadily. A total of 272 species/taxa were identified, of which arthropods were the main dominant group, followed by mollusks and annelids. The number of individuals and species on timber reefs enhanced with oyster shells or leaves/branches exceeds those on simple timber reefs, suggesting that additional materials provide more attachment space for marine organisms. The seasonal variation showed that the number of individuals was higher in summer and lower in winter, but the seasonal variation gradually weakened over time, indicating that the community may tend to mature. The diversity index shows that the diversity of wood reefs with oyster shells and wood reefs with leaves/branches is higher than that of simple wood reefs, indicating that additional materials can provide more complex habitat structures to attract more fish. It can be found from the above that wood artificial reefs with additional materials can not only provide a richer source of food, but also promote the diversity of marine ecosystems.

### 3.2. Shipwreck Artificial Reef

As an accidentally formed artificial reef, shipwrecks provide new habitats for marine organisms. The attached communities (such as corals, sponges, etc.) on the surface of shipwrecks play an important role in energy transfer and ecosystem function [70]. Jimenez et al. [71] investigated the attached biological communities of Touba (sunk by a storm on the way to transport military supplies from Limassol Port to Larnaca Port) and Cricket (sunk as a military shooting training target) wrecks near Cyprus, and discussed the ecological effects of wrecks as artificial reefs, especially the diversity and coverage of coral communities. The Touba shipwreck was sunk in 1974, and the Cricket shipwreck was sunk in 1947. By analyzing the underwater photos of 2010 and 2016, the coverage of corals and other periphytons was calculated using image analysis software. The sea surface temperature and chlorophyll concentration of the study area were analyzed using MODIS/Aqua satellite data to evaluate the impact of environmental conditions on the periphyton community. The coral community composition and coverage of the two shipwrecks were compared, and the impact of time changes on the community structure was analyzed. The results showed that the periphyton communities of the two shipwrecks were mainly composed of sponges, corals, and calcareous algae. Sponges have the highest coverage (about 27%) on the two wrecks, while corals have a coverage of between 7% and 19%. The coral community of the Cricket wreck is more developed than that of Touba, especially in the shelter part of the wreck. Although the age difference between the two shipwrecks is 27 years, the time has little effect on the attached biological community unless a major disturbance event occurs. The location, depth, structure, and surrounding environmental conditions of the shipwreck have an important influence on the formation and development of the attached biological community. Taking the sunken destroyer ‘Brisbane’ off the east coast of Australia as the research object, Walker et al. [72] explored the spatial heterogeneity of the epiphytic community of the sunken ship as an artificial reef in the early colonization stage. The study found that the shipwreck was widely colonized by barnacles, sponges, mosses, and other organisms after one year of submergence, and the community structure showed significant differences on the scale of meters to ten meters. Water depth, surface orientation (vertical/horizontal), and water exposure were the key environmental factors affecting community composition and diversity. The biodiversity on the vertical surface is higher than that on the horizontal surface covered by sediments, and the hidden area in the ship is dominated by polychaetes. Medeiros et al. [73] studied the long-term effects of four hundred-year-old shipwrecks in northeastern Brazil as artificial reefs on the fish diversity of coral reefs in the region. We found that shipwrecks can support higher rare species diversity at the local (α diversity) scale, and their community composition is significantly different from that of adjacent natural reefs (only 57% of species are the same). However, at the regional (β diversity) scale, the wreck community showed high homogenization, and its γ diversity was partly derived from the ‘attraction’ of the surrounding natural reef species, which may lead to the degradation of the natural reef community. Studies have shown that wreck artificial reefs enhance local habitat heterogeneity through their structural complexity and help to enhance regional biodiversity. However, improper management may aggravate the homogenization of regional biodiversity and cannot replace the ecological functions of natural reefs.

### 3.3. Ceramic Artificial Reefs

Traditional artificial reefs have some limitations in environmental friendliness and biocompatibility [74]. Kalam et al. [75] designed two kinds of ceramic artificial reefs: hexagonal column reefs and ceramic rods. Their chemical composition, surface texture, water absorption, mechanical strength, erosion rate, and durability were measured and compared with natural coral fragments. In order to evaluate the effects of ceramic materials on water quality and biological productivity, the reefs were placed in a laboratory aquarium to simulate the shallow sea environment, and the availability of nutrients, the diffusion of toxic chemicals, and the reproduction of plankton (bacteria, diatoms) and other benthic organisms were observed. The results show that the ceramic artificial reef is non-toxic, neutral in pH, has high mechanical strength and durability, and is suitable for long-term use in harsh shallow sea environments. The water absorption of ceramic materials is higher than that of natural coral fragments, indicating that there are a large number of tiny voids inside them, which can provide habitats for bacteria and aquatic microorganisms. The mechanical test and erosion test show that the hardness of the ceramic material is equivalent to that of coral fragments. The structure with a wall thickness of 24 mm can withstand a pressure of about 1031 N and has good static stability. However, its flexural strength (4.5 MPa) is significantly lower than that of coral (13.8 MPa), and the erosion rate is higher under the erosion of sand particles with a particle size of ≥1.2 mm, indicating that its durability under a strong dynamic load environment needs to be improved. Biological analysis showed that ceramic reefs had no negative effect on water quality and microbial reproduction, and bacteria, diatoms and other benthic organisms could be continuously propagated on the surface and pore structure of reefs. Fish shelter behavior tests show that ceramic reefs can provide good shelter for small fish and help them escape predators. Ceramic artificial reefs have good application prospects in shallow sea areas and can provide a growth and shelter environment for small fish. O’Reilly et al. [76] selected terracotta clay to make a cylindrical sample with a diameter of 76 mm and a height of 38 mm, and immersed it in circulating seawater for 78 days to evaluate the formation of biofilm. The experiment was divided into three groups: green sample group (exposed to sunlight seawater pool), gray sample group (indoor seawater pool without light), and dry control group. The biofilm formation ability of different materials was evaluated by measuring the dry weight of biofilm, material porosity, and surface pH value. The results show that red pottery clay has a certain potential in biofilm formation, although its biofilm accumulation is lower than that of diatomite-added concrete. The porosity of terracotta clay is high (average 17.2%), which contributes to the formation of biofilm. However, the surface pH value of terracotta clay increases from acidic (pH 6) to neutral (pH 8) after immersion. Although the biofilm formation ability of terracotta clay is not as good as some concrete materials, its porosity and low cost make it a potential candidate material for artificial reefs. Future research should further optimize the manufacturing process of red pottery clay and evaluate its performance in the long-term marine environment to determine whether it is suitable for large-scale application in artificial reef construction.

### 3.4. Waste Tire Artificial Reef

Waste tires are widely used in the construction of artificial reefs around the world because of their easy access, durability, and large gap space. Collins et al. [16] deployed tire modules and concrete modules in the 12 m-deep waters of Poole Bay. The reef was monitored by diving every two months to observe and collect the biological colonization on the tire surface and compare it with the concrete surface. Monitoring methods include observation, collection of biological samples, and close-range photography. The collected biological samples were analyzed for heavy metals and organic compounds. Through research, it was found that the biological colonization rate on the tire surface was faster, and there was no significant difference in the biodiversity of the tire surface compared with the concrete surface. However, the concentration of zinc in the attached organisms on the tire surface was significantly higher than that on the concrete surface, which was related to the zinc compounds used in the tire manufacturing process. Although the biological colonization on the tire surface is good, the potential chemical release of the tire material still needs further study. The type of tire structure (such as rubber rock and tetrahedron) has a greater effect on biological colonization than the material itself (tire or concrete). Tires are inexpensive and readily available, with a porous structure that provides an ideal habitat and attachment surface for algae, coral, shellfish, and fish, effectively promoting the recovery of local fishery resources. However, in seawater leaching tests, tires release heavy metals like zinc and organic compounds such as polycyclic aromatic hydrocarbons, which may impact local water quality and cause bioaccumulation over time. Additionally, if improperly designed or secured, tires can be washed ashore by storms, leading to secondary pollution and visual degradation [77]. Overall, the use of discarded tires in marine environments holds certain ecological potential. While short-term ecological effects are significant, long-term environmental safety requires further systematic evaluation.

## 4. Effects of Artificial Reefs on Marine Environment

### 4.1. Effects of Artificial Reefs on Fish

As an important tool for marine ecosystem restoration and fishery resource management, artificial reefs have been widely used in many sea areas around the world [78]. With the deterioration of natural habitats and the decline of fishery resources, the deployment of artificial reefs has become a key approach to restoring fish habitats [79]. However, there are significant differences in the effects of different types of artificial reefs on fish communities. The following will describe the effects of wood, concrete, shipwreck, steel and stone artificial reefs on fish communities, and analyze their similarities with natural reefs and their potential in fish habitat restoration.

#### 4.1.1. Effects of Wood Artificial Reefs on Fish

**Figure 10:**
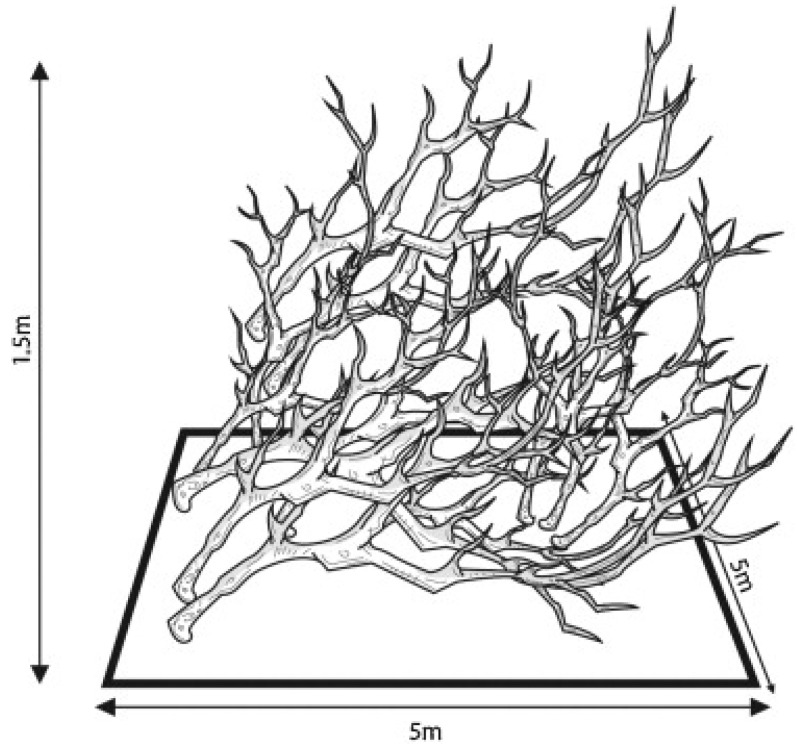
Artificial reef structure diagram, showing the size and location of the branches in the structure [80].

Yamamoto et al. [80] conducted a two-year experiment in four lakes (Canauari Pequeno, Canauari Grande, Prato, and Arraia) in the Anavilianas National Park, Brazil, and constructed eight small artificial reefs with a volume of 37.5 cubic meters. Each reef consists of wooden frames and branches, as shown in Figure 10, to provide a complex habitat.

The control area is an open water area 100 m away from the reef. Fish samples were collected using gillnets in four hydrological cycles (flood period, high water level period, recession period, and low water level period) in 2009 and 2010, and the effects of artificial reefs on fish community composition and diversity were evaluated by thinning curves, Shannon diversity index, and Pielou evenness index. The results showed that the deployment of artificial reefs did not significantly alter the overall fish community diversity index (including Shannon index and Pielou evenness), but the species richness in the reef area was significantly higher than that in the control area. Among the 107 species of fish collected, 69 species were distributed in both reef and control areas, 26 species were only found in reef areas, and 12 species were only found in control areas. Most of the species endemic to the reef area are low-abundance species, which usually do not migrate seasonally and rely on flooded forests as refuges. In the dry season, the unavailability of flooded forests may result in a lower population density of these species. Therefore, artificial reefs may provide an important refuge for these rare and non-migratory species during drought, preventing their local extinction. The application of wood artificial reefs in lakes can be found to have potential protection value, especially in providing habitats for rare species. Alam et al. [81] deployed three types of timber artificial reefs in Mizuno Bay, Hiroshima, Japan: simple timber reefs, timber reefs with oyster shells, and timber reefs with leaves/branches. The model considers the predator–prey relationship and the construction of biological quantity change. The number of fish was recorded by an underwater camera, and the factors such as differentiation and fish fishing were simulated by numerical model, and the model was verified by observation data. The results showed that the timber artificial reefs with additional materials (oyster shells and leaves/branches) were more significant in increasing fish biomass. Compared with simple timber reefs, reefs with oyster shells and leaves/branches provided more habitat space and food sources, attracting more fish. The numerical model shows that the amount of food flow in reefs with additional materials is two to three times that of simple reefs, and the fish biomass increases significantly. The use of by-products of forestry and oyster farming to construct artificial reefs not only helps to increase fishery production, but also promotes the establishment of a circular society. Through the above research, it can be found that the complexity of wood artificial reefs provides shelter for juvenile fish, further promoting the recovery of fish populations and the richness of fish species.

#### 4.1.2. Effect of Concrete–Shipwreck Artificial Reef on Fish

**Figure 11:**
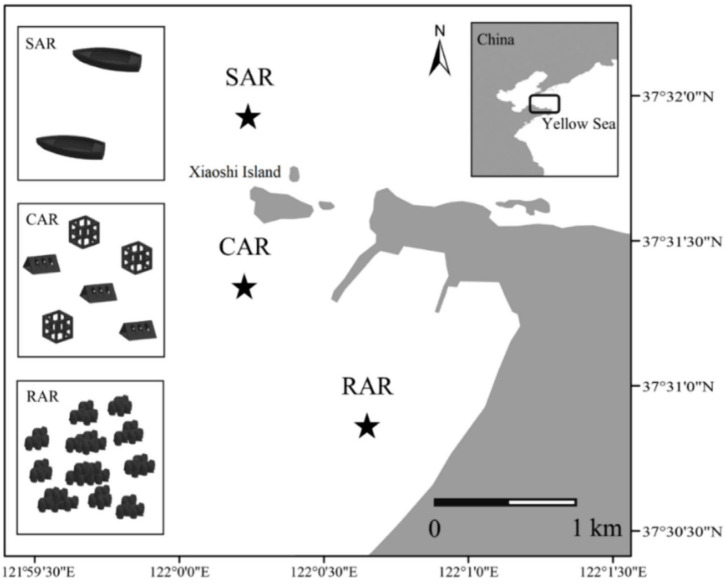
Sampling points of artificial reef habitats (concrete artificial reef, rock artificial reef, and shipwreck artificial reef) around Xiaoshi Island in the northern Yellow Sea, China [83].

Lemoine et al. [82] evaluated the ecological performance of concrete, old ship-modified artificial reefs and natural reefs by diving surveys on 23 artificial and natural reefs off the coast of North Carolina. The abundance, biomass, and species richness, along with community composition of fish, were documented via a 30 m × 4 m belt transect method. The ‘footprint’ and structural complexity of reefs were also measured to analyze the effects of these factors on fish communities. The data were analyzed by a generalized linear mixed model (GLMM) and multivariate statistical methods (such as PERMANOVA and SIMPER) to determine the differences in fish communities among different reef types and their potential driving factors. The results show that concrete artificial reefs are highly similar to natural reefs in fish abundance, biomass, and community composition, indicating that these structures can effectively simulate natural habitats. In contrast, shipwreck reefs support different fish communities, and fish abundance and biomass are significantly higher than concrete reefs and natural reefs. Further analysis showed that the ‘footprint’ and structural complexity of reefs were important factors affecting the differences in fish communities. The structural complexity of concrete reefs is positively correlated with fish abundance, especially after excluding high-complexity shipwreck reefs. Concrete artificial reefs perform well in simulating the function of natural reefs and are suitable for habitat restoration projects. Wreck reefs are more suitable for habitat replenishment projects that increase fish abundance and biomass. Song et al. [83] selected three different types of artificial reef habitats: concrete artificial reef, rock artificial reef, and shipwreck artificial reef as shown in Figure 11.

**Figure 12:**
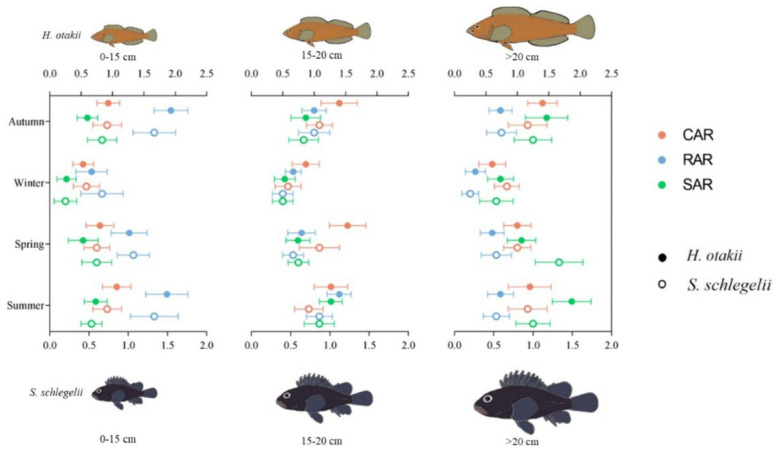
The average abundance of six-line fish and black sea bream in each size class of concrete artificial reef (CAR), rock artificial reef (RAR), and shipwreck artificial reef (SAR). The error line represents the standard error. The abundance unit is expressed as the num [83].

In this study, the feeding ecology and trophic niche differentiation of two representative rock reef fish, Hexagrammos and Sparus macrocephalus, in different habitats were analyzed by using the method of gastric content analysis and stable isotope technology. The two fishes are both dominant settled rock reef fishes in the artificial reef area of the northern Yellow Sea, with weak activity and strong dependence on local habitats. Fish samples were divided into three size groups (0–15 cm, 15–20 cm, >20 cm) according to body length, and were sampled in different seasons (spring, summer, autumn, and winter). Through the analysis of stomach contents, the composition and diversity of fish feeding were determined. The trophic source and trophic width of fish were evaluated by stable isotope analysis. The results showed that different types of artificial reef habitats had significant effects on the feeding ecology and trophic niche differentiation of Hexagrammos and Sparus macrocephalus. The analysis of gastric contents showed that the feeding diversity of the six-line fish was higher than that of the black sea bream, and the feeding diversity of the two fish in the concrete artificial reef was the highest. Stable isotope analysis further confirmed that there were significant differences in the nutritional sources of fish in different habitats. The trophic breadth of fish in concrete artificial reefs was the largest, indicating that the habitat could provide a wider range of feeding options. The study also found that there were significant differences in the feeding strategies of fish in different size groups in different habitats. Small fish had higher feeding diversity in rock artificial reefs, while large fish had higher feeding diversity in hull artificial reefs, as shown in Figure 12.

These results indicate that each artificial reef habitat can provide sufficient resources for fish, but there are significant spatial and interspecific differences in feeding behavior and trophic differentiation in different habitats. The artificial reef project in Turkey by Altan et al. [84] mainly adopts reinforced concrete modules with various designs, including cubes, plus-shaped, and pentagonal domes. Monitoring research is mainly carried out by divers using visual census technology to record fish species, quantity, and the presence of large invertebrates. The specific experimental site was Hekim Island, and the monitoring time ranged from 1 year to 8 years after the deployment of the reef. The study found that in the Hekim Island project, the number of fish species increased from 8 to 16 after one year of reef deployment, and further increased to 22 after 8 years. The number of fish also increased from 134 ± 25 to 259 ± 56, showing a significant increase (*t* test, *p* < 0.05). This indicates that artificial reefs effectively attract a variety of fish and promote their increase in number. Reefs at depths of 9 m and 18 m increase in fish species and number in summer and decrease in winter. This seasonal change is related to fish migration behavior, further confirming the importance of artificial reefs to fish habitats.

#### 4.1.3. Effects of Steel Artificial Reefs on Fish

**Figure 13:**
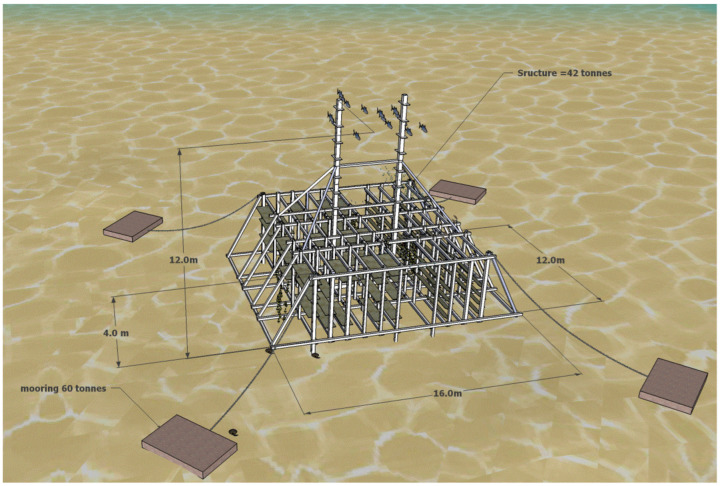
The structure and size of artificial reefs [85].

Becker et al. [85] evaluated the impact of artificial reefs off the coast of Sydney on fish communities, and used stereo-BRUV and SeaViewer TM underwater cameras to monitor reef-related fish and pelagic fish, respectively. In order to compare the differences between artificial reefs and natural reefs, three natural reefs were selected as control points. Statistical methods such as PERMANOVA (permutation multivariate analysis of variance) and SIMPER (similarity percentage analysis) were used to assess the interannual variability and species diversity of fish communities. The results showed that the fish community of artificial reefs was significantly different from that of natural reefs, and it showed obvious interannual variation. A total of 53 fish species were observed in the artificial reefs, comprising various key fishery species and their prey. Although the fish community of artificial reefs is different from that of natural reefs, it has successfully provided habitats for a variety of fish, especially for juvenile and adult fish. In addition, the design of artificial reefs (such as high-rise tower structures) provides a habitat for upper-middle fishes and increases the diversity of fish communities, as shown in Figure 13.

The fish community of artificial reefs did not converge with that of natural reefs within four years, indicating that long-term monitoring is essential for understanding the ecological effects of artificial reefs. The Sydney offshore artificial reef has successfully achieved its goal, providing high-quality fishery resources for anglers and providing an important reference for future reef design and deployment.

### 4.2. Effects of Artificial Reefs on Zoobenthos Community

As an important tool for marine ecosystem restoration and fishery resources restoration, artificial reefs have been widely used in coastal areas around the world [86]. Different types of reefs may have different effects on the surrounding macroinvertebrate communities due to differences in their structure, materials, and layout [87]. Benthic fauna is a key biological group in marine sediments, usually between 500 microns and 31 microns [88,89], with high diversity, high abundance, and short life cycle. They play an important role in marine ecosystems. They are not only an important link in the food chain, but also participate in the decomposition of organic matter and the circulation of nutrients [90,91]. However, the study on the mechanism of different types of artificial reefs on benthic communities, especially the use of high-throughput sequencing technology, is still limited. The effects of concrete, shipwrecks, and rock artificial reefs on mesobenthos communities are analyzed below, and their response mechanisms at different seasons and spatial scales are discussed.

#### 4.2.1. Effects of Concrete Artificial Reefs on Macroinvertebrate Communities

**Figure 14:**
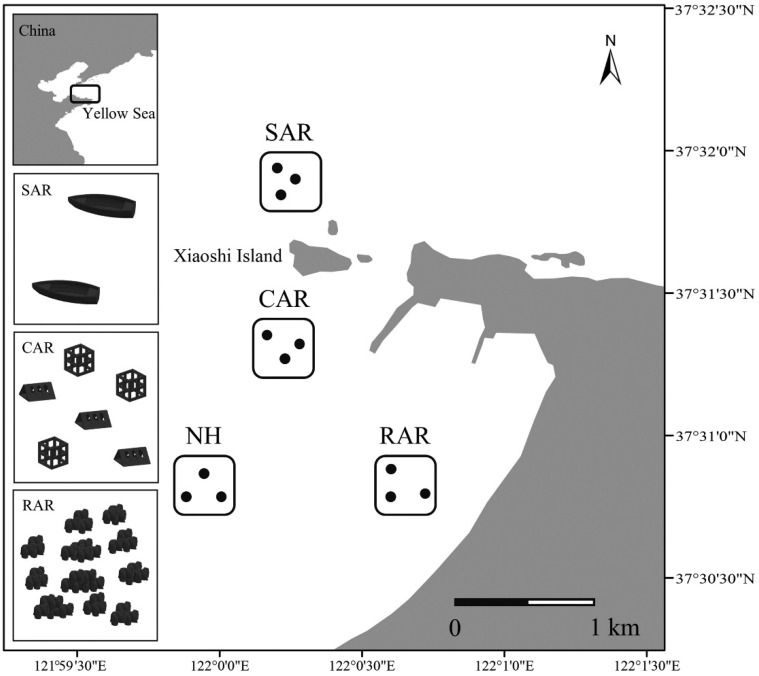
The artificial reef habitats around Xiaoshi Island include concrete artificial reef (CAR), rock artificial reef (RAR), shipwreck artificial reef (SAR), and natural habitat (NH) sampling points [92].

Song et al. [92] conducted a study in the Xiaoshidao area of the northern Yellow Sea, China, and selected concrete artificial reefs, rock artificial reefs, shipwreck artificial reefs, and adjacent natural habitats as research objects. The diversity and community structure of zoobenthos in different habitats were analyzed by high-throughput sequencing of 18 S rRNA gene. Sediment samples were collected in the winter, spring, summer, and autumn of 2021, respectively. Each habitat has three sampling points as shown in Figure 14.

**Figure 15:**
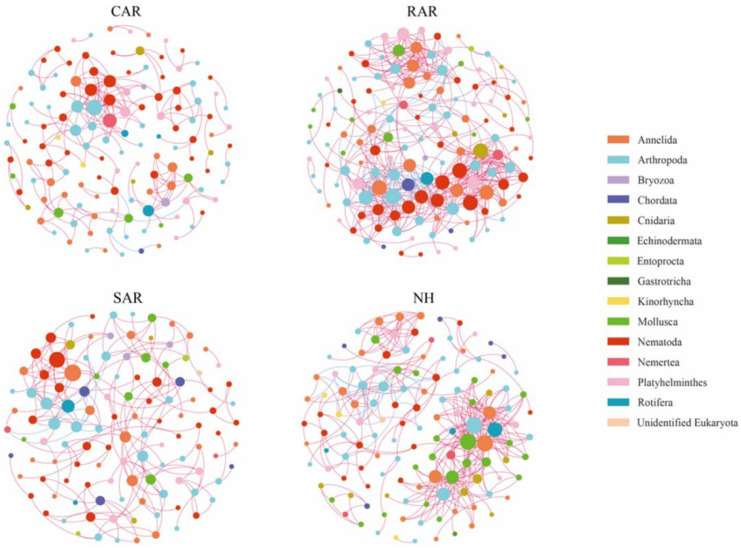
Co-occurrence network analysis of small and medium-sized animal communities at the genus level in CAR, RAR, SAR, and NH. The color of the node represents different gates and the size of the node is proportional to the number of connections. Each connection represents a strong (|r| > 0.7) and significant (*p* < 0.05) correlation, with red indicating a positive correlation and blue indicating a negative correlation [92].

The flow field effects of different types of artificial reefs were simulated by computational fluid dynamics method, and their effects on sediment environmental parameters were analyzed. Environmental parameters include water temperature, dissolved oxygen, salinity, pH, total organic carbon, and sediment particle size. The association between environmental variables and benthic communities was examined using redundancy analysis and the Mantel test. The results demonstrated that the concrete artificial reefs substantially altered the properties of adjacent sediments, particularly sediment particle size and total organic carbon content. The sediment grain size of the concrete artificial reef area is similar to that of the natural habitat, but the total organic carbon content is significantly higher than that of the natural habitat. The diversity and community structure of benthic communities showed significant differences in spatial and temporal scales. The diversity index (Shannon index and Pielou index) of benthic animals in the concrete artificial reef area was significantly lower in summer and autumn than in winter and spring, and the diversity index of the area was higher than that of the shipwreck artificial reef and natural habitat, but lower than that of the rock artificial reef. The analysis of the macroinvertebrate community structure showed that the macroinvertebrate community in the concrete artificial reef area was similar to that in the wreck artificial reef area in winter and spring, but showed significant differences in summer and autumn. Dominant group analysis and co-occurrence network analysis showed that the macroinvertebrate community in the concrete artificial reef area was dominated by arthropods, followed by link and flat, and the macroinvertebrate community network had a high modularity coefficient, indicating that the community structure was relatively stable as shown in Figure 15.

**Figure 16:**
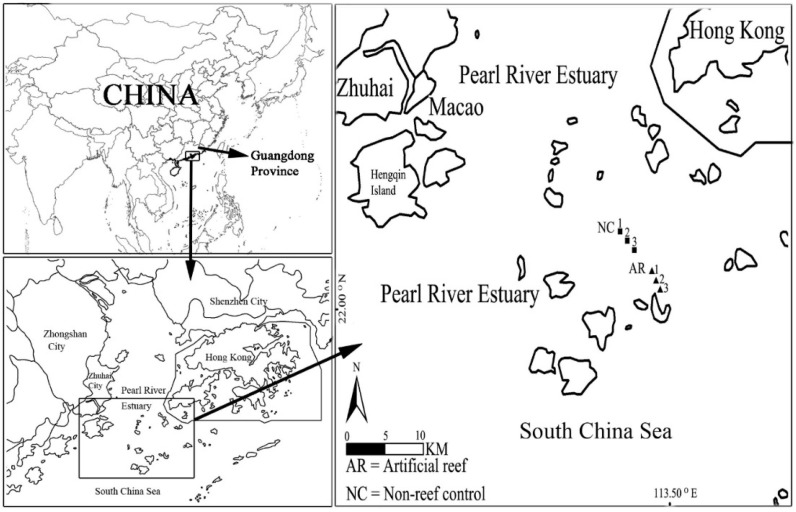
The location and sampling points (1–3) of artificial reefs in the Pearl River Estuary, China. Three artificial reef sampling sites are located on coral reefs (▲), and three non-reef control points are close to but not on coral reefs (■) [93].

Chen et al. [93] conducted a two-year survey in the Pearl River Estuary. Before the construction of the concrete artificial reef (2010) and after the construction (2012), the benthic community was sampled in the artificial reef habitat and the nearby non-reef control habitat. The sampling time was March, May, August, and November, and three sampling points were selected for each habitat, as shown in Figure 16.

Benthic animal samples were collected by a 0.1-square-meter benthic sampler and screened using a 1.0 mm sieve. After the samples were preserved in alcohol, the species identification and biomass determination were carried out under the microscope. By calculating the Shannon–Weiner diversity index, Simpson diversity index, Margalef species richness index, Pielou evenness index, and other taxonomic diversity indexes, the structural changes in zoobenthos communities were evaluated. The study also analyzed the thermodynamic health status of benthic communities through the calculation of ecological energy and specific ecological energy. The results showed that after the construction of artificial reefs, the diversity, species richness, and biomass of benthic animals showed an increasing trend in both artificial reef habitats and nearby non-reef control habitats. The change in ecological energy was positively correlated with the biomass and abundance of benthic animals, indicating that ecological energy mainly came from biomass information. The construction of artificial reefs did not change the composition of dominant species in benthic communities, but changed their dominance values. The study also found that when using two different ecological exergy calculation methods (SWF and CV) to calculate the ecological energy value, the ecological energy value obtained by the CV method is significantly higher than that of the SWF method, which may be related to the difference in biological information contained in the two methods. Overall, the construction of artificial reefs has a positive impact on the taxonomic diversity and thermodynamic health of benthic communities in the short term.

#### 4.2.2. Effects of Rock Artificial Reefs on Zoobenthos Community

Burt et al. [94] selected six rock breakwaters ranging in age from 1 to 31 years in the southeastern basin of Dubai’s Persian Gulf and compared them with benthic communities on natural reefs. By sampling using the photo sample method, through the 30 m long sample line every 3 m to take 0.25 square meters of sample photos, a total of 66 photos were taken. The CPCe image analysis software (v4.0) was used to classify and analyze the benthic organisms in each photo. Multivariate statistical methods such as non-metric multidimensional scaling and similarity analysis were used to compare the differences in benthic communities on breakwaters and natural reefs of different ages. The results showed that the benthic community on the breakwater gradually approached the community on the natural reef with age, but even the most mature breakwater’s (31 years) community was still significantly different from the natural reef. Young breakwaters (<5.5 years) are dominated by algae, sponges, bivalves, and other organisms, while more mature breakwaters (≥25 years) are dominated by corals. The coral coverage on the 25-year and 31-year breakwaters was significantly higher than that on natural reefs. These results indicate that the benthic community on the breakwater has continued to change for more than 31 years. Although it gradually approaches the natural reef community with age, it still remains unique. The study highlights the importance of artificial reefs in providing a rich and unique habitat for benthic communities and suggests that these structures should be constructed in as ecologically sensitive a manner as possible to protect tropical natural reef habitats.

#### 4.2.3. Effects of Shipwreck Artificial Reefs on Benthic Fauna Community

Pinto et al. [95] selected two shipwrecks on the east coast of Brazil as research objects, located in exposed and sheltered areas, respectively. The study area also includes natural reefs 0, 1, and 2 km away from the wreck. A 50 × 50 cm quadrat photograph was taken by diving to record the coverage of benthic organisms. PhotoQuad software (v1.0) was used to analyze the photos and calculate the percentage coverage of benthic organisms in each quadrat. All invertebrates were identified to the lowest classification level as far as possible, and algae were classified according to functional groups. To mitigate the impact of depth variations on the community, principal component analysis served to establish a spatial filter. Subsequently, non-metric multidimensional scale analysis and permutation multivariate analysis of variance were utilized to compare community similarities and differences across different reefs. The results showed that the benthic community structure of the shipwreck artificial reef and the nearby natural reef within 1 km showed high similarity in the exposed area. However, the community structure of the shipwreck and the natural reef in the sheltered area was significantly different and not affected by the distance. The high wave energy in the exposed area promoted the diffusion of larvae and the homogenization of the community, while the low wave energy in the sheltered area increased the heterogeneity of the community. The wreck artificial reef can simulate the benthic community structure of natural reefs in the exposed area, but it shows obvious differences in the sheltered area. Therefore, when introducing artificial reefs, local hydrodynamic conditions and distance from the natural environment should be considered to reduce the negative impact on the natural environment.

## 5. Economic Analysis

The deployment of artificial reefs not only aims to restore marine ecosystems, but also hopes to improve the livelihoods of coastal fishermen by increasing fish resources. Sapehee et al. [96] conducted a questionnaire survey on 172 fishermen in Tenggara, Malaysia, and collected data on their cognition and use of artificial reef projects and their impact on household income. Through research, it is found that the deployment of artificial reefs has significantly increased the income level of fishermen and promoted the diversification of income. Among the 172 fishermen surveyed, 90.7% of fishermen have a high awareness of the existence of artificial reefs and 58.1% of fishermen actively use artificial reefs in fishing activities. Artificial reefs increase fish resources, reduce fishing workload, increase the value of catches, and improve the socio-economic status of fishermen. The ‘spillover effect’ of artificial reefs also benefits the adjacent fishing areas and further promotes the sustainable development of fisheries. Whitmarsh et al. [97] analyzed the economic benefits of the artificial reef system in the Algarve, Portugal. The data included the unit effort catch (CPUE) and the market price of each fish, which was used to calculate the unit effort value (VPUE). In fishery science and resource economics, CPUE and VPUE are two commonly used indicators to measure the efficiency and economic benefits of fishery activities. Through multiple regression models, the researchers evaluated the effects of artificial reefs, reef types, locations, and time on VPUE. Regression models include linear models and logarithmic linear models to capture the impact of different variables on economic benefits. The results showed that the VPUE of artificial reefs was about EUR 13 higher than that of the control sites, and the difference was statistically significant (*p* = 0.011). In addition, the time interaction coefficient in the model shows that the VPUE of the artificial reef area increases with time, with an average monthly increase of 0.177 euros, indicating that the ecological and economic benefits of the reef gradually increase with the establishment time. The log–linear model further confirmed these findings, indicating that the VPUE of the artificial reef was 1.73 times that of the control site at the initial stage of installation, and the VPUE increased by 1.4 times over time. Edi et al. [98] analyzed the economic and ecological potential benefits of artificial reef cultivation activities in Indonesia. It is estimated that about 9 square meters of planted artificial reefs can produce a total economic value of about 4.58 million rupees per year, mainly including direct benefits (such as ornamental fish production) and indirect benefits (such as providing fish habitats, coastal protection, and carbon sinks). Although these benefits are not directly reflected in the significant increase in fishermen’s income, they maintain the fishery production potential through ecological functions. At the same time, by introducing local community groups to participate in construction and management, the project has enhanced the community’s sense of participation in resource protection and laid the foundation for its long-term ecological and economic resilience. It is worth noting that under the open fishing conditions without effective supervision, artificial reefs can accumulate fish and improve catch accessibility in the short term, but it is easy to cause overfishing, resulting in a decline in long-term catches, which is not conducive to the sustainable development of community fishery economy. The case of Senegal shows that fishermen are prone to conflicts due to uneven fishing opportunities, and young fishermen are more inclined to support open fishing due to livelihood pressure, exacerbating resource pressure [99]. In summary, it can be found that the economic benefits of artificial reefs show obvious conditional dependence. In areas with effective supervision and high community participation, it can significantly increase fishery income, promote catch appreciation, and produce sustainable ‘spillover effects’. However, if there is a lack of reasonable access rules and long-term supervision, it is easy to induce overfishing, resource competition, and long-term income decline. Therefore, its success depends not only on the ability of ecological restoration, but also on the supporting social governance system and community co-management mechanism.

## 6. Conclusions

Artificial reef is an effective tool for marine ecological restoration and fishery resource management. Its material selection and performance optimization are the key to balancing ecological benefits, engineering durability, and environmental sustainability. First of all, in terms of material development and performance, it is an effective way to improve the mechanical properties and environmental compatibility of concrete reefs by using industrial solid waste (such as steel slag and blast furnace slag) and special cement (such as sulfoaluminate cement) to partially or completely replace traditional Portland cement. This kind of material can not only realize the resource utilization of solid waste and reduce the environmental load, but also its optimized hydration products and microstructure can usually bring higher compressive strength and durability. At the same time, it helps to make the surface microenvironment of the reef more neutral, thus promoting the early attachment and colonization of marine organisms. In contrast, some concrete filled with organic waste (such as banana peel) or biological shell (such as oyster shell) may not be as strong as traditional concrete in terms of absolute strength, but it shows unique potential in providing biological nutrients, enhancing chemical erosion resistance, and promoting the formation of specific biological communities, providing new ideas for functional reef design.

In terms of ecological benefits, whether using traditional or new materials, the deployment of artificial reefs has been widely confirmed to significantly increase the habitat complexity of local sea areas, thereby increasing species abundance, biodiversity, and biomass. This ecological aggregation effect is the basis for restoring fishery resources and improving the structure and function of marine ecosystems. Different materials may attract and carry different biological communities due to their differences in surface physical and chemical properties, structural morphology and degradation characteristics, which provides a basis for the selection of reef materials for specific ecological restoration objectives (such as proliferation of specific species and the restoration of seagrass beds or coral reefs).

From the perspective of resources and environment, promoting the ‘greening’ and ‘waste recycling’ of artificial reef materials is an important direction for future development. A large number of studies have shown that the use of industrial, construction, and agricultural waste for reef preparation is technically feasible and can produce significant environmental and economic synergies. This not only reduces the pressure on waste disposal, but also reduces the cost of raw materials for reefs, in line with the principles of circular economy.

At the socio-economic level, artificial reefs have been shown to have a positive impact on the income of fishermen in coastal communities by repairing fishery resources and creating new fishing grounds, which is an important means to promote ‘blue growth’ and sustainable livelihoods.

In summary, the future research and practice of artificial reefs should focus on the following: (1) deepening the basic research of material science, further optimizing the solid waste-based and eco-friendly cementitious material system and developing standardized and modular high-performance prefabrication technology; (2) strengthening long-term ecological monitoring and impact assessment and quantifying the ecological service function of different material reefs in the whole life cycle and their long-term and comprehensive impact on the ecosystem through multidisciplinary means; and (3) promoting life cycle assessment and cost-benefit analysis and providing decision makers with comprehensive decision support from environmental to economic to social benefits. Through the collaborative progress of material innovation, ecological cognition, and management strategy, artificial reefs will play a more central role in the construction of marine ecological civilization and the sustainable utilization of fishery resources.

## Figures and Tables

**Table 1:** Mix proportion of artificial reef concrete [51].

Sample ID	OPC	SAC	Sea Sand	River Sand	Sea Water	Fresh Water	Stone	SP	Retarder
S0	515	0	0	545	0	165	1270	7.21	1.55
S1	0	515	0	545	0	165	1270	7.21	1.55
S2	0	515	0	545	165	0	1270	7.21	1.55
S3	0	515	545	0	0	165	1270	7.21	1.55
S4	0	515	545	0	165	0	1270	7.21	1.55

OPC: Portland cement; SAC: sulfoaluminate cement; SP: superplasticizer.

**Table 2:** Mix proportion of artificial reef concrete [56].

Sample ID	OPC	SAC	Sea Sand	River Sand	Sea Water	Fresh Water	Stone	SP	Retarder
OFR	368	0	0	640	0	184	1242	3.68	0
SFM	0	368	640	0	0	184	1242	3.68	1.104
SSR	0	368	0	640	184	0	1242	3.68	1.104
OSM	368	0	640	0	184	0	1242	3.68	0
OSSM	184	184	640	0	184	0	1242	3.68	0.552
SSM	0	368	640	0	184	0	1242	3.68	1.104

OPC: Portland cement; SAC: sulfoaluminate cement; SP: superplasticizer.

**Table 3:** Mix proportion of artificial reef concrete [58,59].

Sample ID	OPC	SAC	Sea Sand	River Sand	Sea Water	Fresh Water	Stone	SP	Retarder
OFR	368	0	0	640	0	184	1242	3.68	0
OSM	368	0	640	0	184	0	1242	3.68	0
OSSM	184	184	640	0	184	0	1242	3.68	0.552
SSM	0	368	640	0	184	0	1242	3.68	1.104
OPCC	515	0	0	545	0	165	1270	7.21	1.55
NARC	0	515	545	0	165	0	1270	7.21	1.55

OPC: Portland cement; SAC: sulfoaluminate cement; SP: superplasticizer.

## Data Availability

No new data were created or analyzed in this study. Data sharing is not applicable to this article.
